# MicroRNA-155 Promotes Autophagy to Eliminate Intracellular Mycobacteria by Targeting Rheb

**DOI:** 10.1371/journal.ppat.1003697

**Published:** 2013-10-10

**Authors:** Jinli Wang, Kun Yang, Lin Zhou, MinhaoWu, Yongjian Wu, Min Zhu, XiaoMin Lai, Tao Chen, Lianqiang Feng, Meiyu Li, Chunyu Huang, Qiu Zhong, Xi Huang

**Affiliations:** 1 Department of Immunology, Institute of Tuberculosis Control, Institute of Human Virology, Zhongshan School of Medicine, Sun Yat-sen University, Guangzhou, China; 2 Key Laboratory of Tropical Diseases Control (Sun Yat-sen University), Ministry of Education, Guangzhou, China; 3 Center for Tuberculosis Control of Guangdong Province, Guangzhou, China; University of New Mexico, United States of America

## Abstract

*Mycobacterium tuberculosis* is a hard-to-eradicate intracellular pathogen that infects one-third of the global population. It can live within macrophages owning to its ability to arrest phagolysosome biogenesis. Autophagy has recently been identified as an effective way to control the intracellular mycobacteria by enhancing phagosome maturation. In the present study, we demonstrate a novel role of miR-155 in regulating the autophagy-mediated anti-mycobacterial response. Both *in vivo* and *in vitro* studies showed that miR-155 expression was significantly enhanced after mycobacterial infection. Forced expression of miR-155 accelerated the autophagic response in macrophages, thus promoting the maturation of mycobacterial phagosomes and decreasing the survival rate of intracellular mycobacteria, while transfection with miR-155 inhibitor increased mycobacterial survival. However, macrophage-mediated mycobacterial phagocytosis was not affected after miR-155 overexpression or inhibition. Furthermore, blocking autophagy with specific inhibitor 3-methyladenine or silencing of autophagy related gene 7 (Atg7) reduced the ability of miR-155 to promote autophagy and mycobacterial elimination. More importantly, our study demonstrated that miR-155 bound to the 3′-untranslated region of Ras homologue enriched in brain (Rheb), a negative regulator of autophagy, accelerated the process of autophagy and sequential killing of intracellular mycobacteria by suppressing Rheb expression. Our results reveal a novel role of miR-155 in regulating autophagy-mediated mycobacterial elimination by targeting Rheb, and provide potential targets for clinical treatment.

## Author Summary

microRNA-155 (miR-155) plays an essential role in regulating the host immune response by post-transcriptionally repressing the expression of target genes. However, little is known regarding its activity in modulating autophagy, an important host defense mechanism against intracellular bacterial infection. *Mycobacterium tuberculosis* is a hard-to-eradicate intracellular pathogen that infects approximately one-third of the global population, and causes 1.5 million deaths annually. The present study explores a novel role of miR-155 in the host response against mycobacterial infection. Our data demonstrates that mycobacterial infection triggers the expression of miR-155, and the induction of miR-155 in turn activates autophagy by targeting Rheb, a negative regulator of autophagy. miR-155-promoted autophagy accelerates the maturation of the mycobacterial phagosome, thus decreasing the survival of intracellular mycobacteria in macrophages. These findings contribute to a better understanding of the host defense mechanisms against mycobacterial infection, providing useful information for development of potential therapeutic interventions against tuberculosis.

## Introduction

*Mycobacterium tuberculosis* (*M. tuberculosis*) is a hard-to-eradicate intracellular pathogen [1] that infects approximately one-third of the global population, and causes 1.5 million deaths annually [2]. However, only 10% of latent infections lead to active tuberculosis, indicating the importance of host immune defense against mycobacterial infection [2]. The anti-mycobacterial immune system is mainly dependent on cellular immunity mediated by macrophages and T lymphocytes [2]. Macrophages belong to the first line of anti-mycobacterial immune defense and recognize the invading mycobacteria by virtue of various pattern recognition receptors (PRRs) [3]. Macrophages also function to secrete inflammatory cytokines and to present bacterial peptide to T lymphocytes, thus leading to a rapid activation of the adaptive immune response [3]. Although a host can deploy a multitude of immune defense mechanisms against *M. tuberculosis*, the bacteria is capable of surviving and persisting within host macrophages because of its repertoire of evading the host immune response [4]. For instance, *M. tuberculosis* can limit the acidification and maturation of mycobacterial phagosomes to escape degradation by lysosomal hydrolases, preventing subsequent antigen presentation [5], [6]. In turn, the host also evolves unique ways to combat intracellular pathogens, such as initiating autophagy to reverse the mycobacteria-induced inhibition of phagosome maturation [7], [8].

Autophagy is an evolutionarily conserved process which is involved in maintaining cytoplasmic homeostasis by degrading damaged organelles or misfolded proteins [9], [10]. The autophagic cascade is initiated by the engulfment of cytoplasmic cargoes by an autophagosome, which then fuses with a late endosome to form the autolysosome, exposing the inner compartment to lysosomal hydrolases for degradation [11]. Several markers of autophagy are well characterized, such as autophagy-related gene 7 (Atg7) and microtubule-associated protein light chain 3 (LC3). Atg7 acts as an E1-activating enzyme taking part in membrane elongation, while LC3 undergoes conversion of LC3-I into its lipidated form LC3-II and is specifically located on the autophagosome until being degraded during autolysosome maturation [12]. Accumulating evidence demonstrates that autophagy is a crucial defense mechanism against a variety of intracellular pathogens, including *Shigella flexneri* [13], *Salmonella typhimurium* [14], *Listeria monocytogenes* [15] and *M. tuberculosis* [7]. Induction of autophagy overcomes the trafficking block imposed by *M. tuberculosis*, thus increasing the acidification and maturation of mycobacterial phagosomes, and inhibiting mycobacterial survival in macrophages [7].

Previous studies have demonstrated that autophagy can be activated by physiological signals (e.g., starvation), pharmacological agonists (e.g., rapamycin), or immunological stimuli, such as TLR ligands and cytokines (e.g., IFN-γ and TNF) [12]. The mammalian target of rapamycin (mTOR) plays a key role in inhibiting autophagy [16], and its activity is enhanced by Ras homologue enriched in brain (Rheb) [17]. Activation of autophagy is essential for initiating a protective response and adapting cells to metabolic stresses and immunological challenges [18]. However, excessive autophagic activation, derailed autophagic trafficking or imbalanced degradation may produce pathogenic conditions, leading to cellular toxicity and death [19]. Therefore, autophagy must be tightly regulated. Although signaling pathways governing autophagy remain to be fully delineated, recent evidence suggests that several microRNAs (miRNAs) may participate in modulating autophagy by directly targeting autophagy-related genes, such as Beclin1 and Atg4 [20].

As a class of small non-coding RNAs, miRNAs are highly conserved between different eukaryotic species and function as key regulators of gene expression at the post-transcriptional level by targeting mRNAs for translational repression or degradation [21]. It is reported that several miRNAs fine-tune the innate and adaptive immune responses to mycobacterial infection. For example, miR-125b decreases TNF production by directly targeting its 3′UTR, whereas miR-155 enhances TNF production by increasing TNF mRNA half-life [22]. miR-144* expression is elevated in active TB patients and functions to inhibit TNF and IFN-γ production and T cell proliferation [23]. miR-29 suppresses IFN-γ production in natural killer cells, and CD4+ and CD8+ T cells by directly targeting IFN-γ mRNA, thus contributing to the host susceptibility to mycobacterial infection [24]. However, the role of miRNAs in autophagy-mediated intracellular bacterial elimination remains unclear.

In the present study, we investigated the potential role of miR-155 in modulating autophagy and bacterial clearance in macrophages. Our study demonstrated that miR-155 expression was significantly induced after mycobacterial infection *in vivo* and *in vitro*. Overexpression of miR-155 promoted autophagy and the maturation of mycobacterial phagosomes in macrophages, thus facilitating the elimination of intracellular mycobacteria. More importantly, we identified Rheb as a novel functional target of miR-155 in eliminating intracellular mycobacteria. These findings provide a better understanding of host defense mechanisms in mycobacterial infection.

## Results

### miR-155 expression is significantly increased *in vivo* and *in vitro* after mycobacterial infection

**Figure 1:**
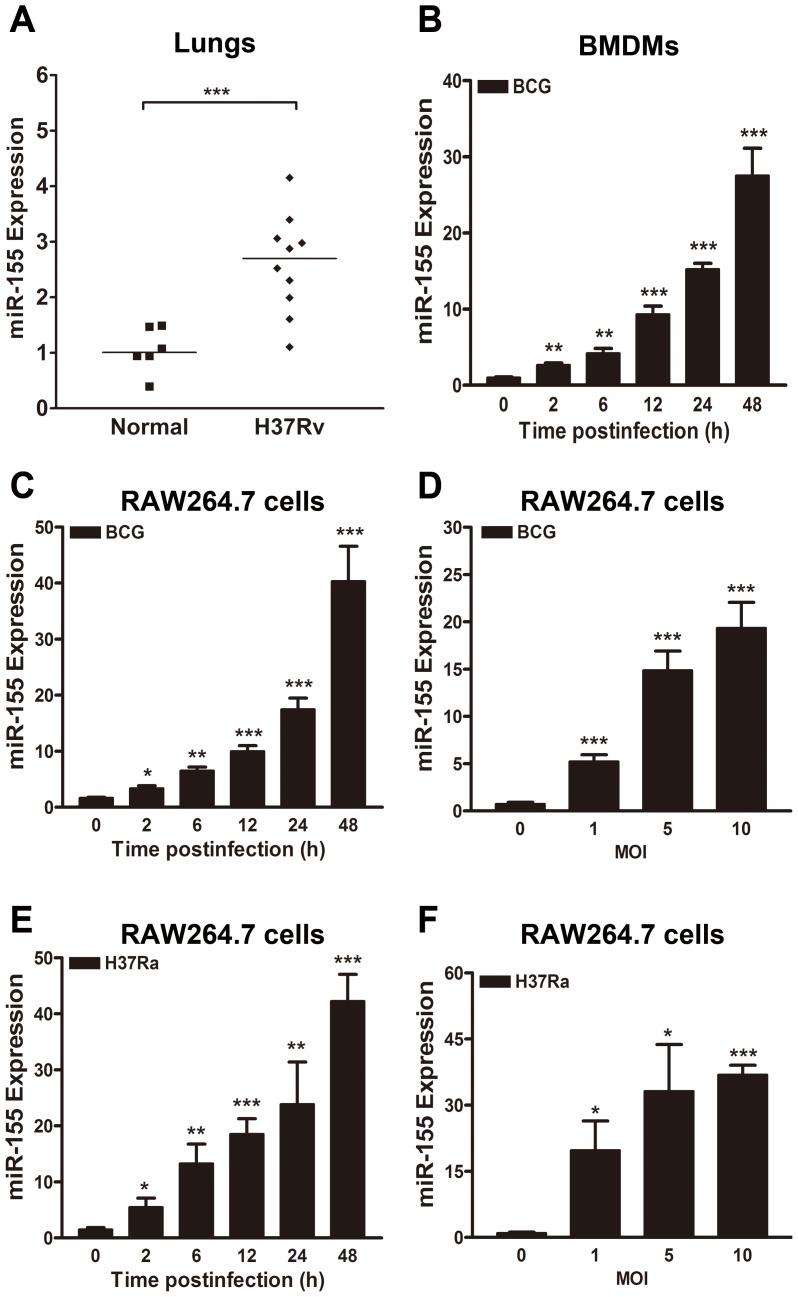
miR-155 expression is induced after mycobacterial infection. (A) miR-155 expression levels were examined in the lungs of normal uninfected or H37Rv infected BALB/c mice 6 weeks postinfection. (B) Murine bone marrow-derived macrophages (BMDMs) were infected with BCG at an MOI of 5 for the indicated times, and the expression levels of miR-155 were measured by real-time PCR. (C and D) RAW264.7 cells were infected with BCG at an MOI of 5 for the indicated time points (C) or at indicated MOIs for 24 h (D). The expression levels of miR-155 were examined by real-time PCR. (E and F) RAW264.7 cells were infected with H37Ra at an MOI of 5 for the indicated time (E) or at indicated MOI for 24 h (F). The expression levels of miR-155 were examined by real-time PCR. Data are shown as the mean ± SEM of three independent experiments. *, p<0.05; **, p<0.01; ***, p<0.001.

To determine the expression level of miR-155 *in vivo* in response to mycobacterial infection, miR-155 expression was tested in the lungs of *M. tuberculosis* H37Rv (H37Rv) infected-BALB/c mice. miR-155 expression in the lungs of H37Rv-infected mice was approximately 2.5-fold higher than in normal uninfected animals (Fig. 1A, p<0.001). We further examined mycobacteria-induced miR-155 induction in cultured murine bone marrow-derived macrophages (BMDMs) and macrophage-like RAW264.7 cells. miR-155 expression was increased in *M. bovis* BCG (BCG)-challenged murine BMDMs in a time-dependent manner (Fig. 1B). Moreover, in RAW264.7 cells, miR-155 expression was gradually increased by BCG and *M. tuberculosis* H37Ra (H37Ra) infection in a time- and dose-dependent manner (Fig. 1, C–F).

### miR-155 decreases the survival of intracellular mycobacteria by promoting the maturation of mycobacterial phagosomes

**Figure 2:**
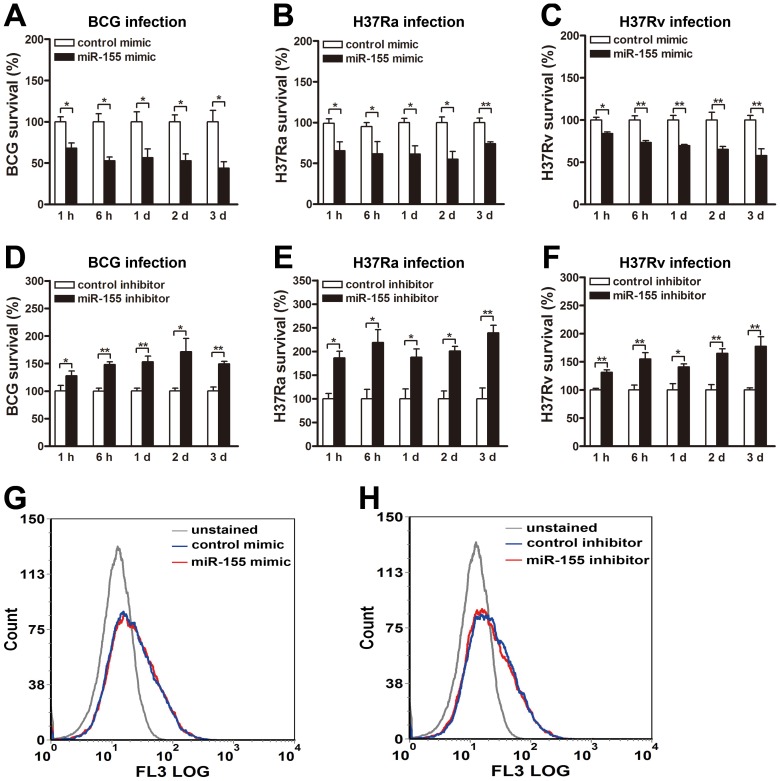
miR-155 decreases mycobacterial survival in macrophages. (A–C) RAW264.7 cells were transfected with control or miR-155 mimic for 24 h followed by BCG (A), H37Ra (B) or H37Rv (C) infection at an MOI of 10 for 1 h, and intracellular mycobacterial viability was determined by CFU assay at the indicated timepoints. (D–F) RAW264.7 cells were transfected with control or miR-155 inhibitor for 24 h followed by BCG (D), H37Ra (E) or H37Rv (F) infection at an MOI of 10 for 1 h, and intracellular mycobacterial viability was determined by CFU assay at the indicated timepoints. (G and H) RAW264.7 cells were transfected with miR-155 mimic (G) or inhibitor (H) for 24 h followed by Texas Red-labeled BCG infection at an MOI of 10 for 1 h. Phagocytosis of mycobacteria was determined by flow cytometry. Data are shown as the mean ± SEM of three independent experiments. *, p<0.05; **, p<0.01.

To determine the role of miR-155 during mycobacterial infection, we next examined its effects on mycobacterial survival by colony-forming unit (CFU) assay. RAW264.7 cells were transiently transfected with miR-155 mimic or inhibitor, and then challenged with BCG, H37Ra or H37Rv at an MOI of 10. Our results showed that miR-155 significantly reduced the survival of intracellular BCG in RAW264.7 cells at 1 h, 6 h, 1 d, 2 d and 3 d postinfection (Fig. 2A, all p<0.05). Moreover, the survival of intracellular H37Ra (Fig. 2B) and H37Rv (Fig. 2C) in RAW264.7 cells transfected with miR-155 mimic were also decreased at the indicated timepoints postinfection (H37Ra: p<0.01 at 3 d postinfection, and p<0.05 at other timepoints; H37Rv: p<0.05 at 1 h postinfection, and p<0.01 at other timepoints).

Additionally, transfection with miR-155 inhibitor increased bacterial survival of BCG in RAW264.7 cells at 1 h, 6 h, 1 d, 2 d and 3 d postinfection (Fig. 2D, p<0.05 at 1 h and 2 d postinfection, and p<0.01 at other timepoints). Similarly, the survival rate of H37Ra (Fig. 2E) and H37Rv (Fig. 2F) also was enhanced in miR-155 inhibitor-treated RAW264.7 cells at the above indicated timepoints postinfection (H37Ra: p<0.01 at 3 d postinfection, and p<0.05 at other timepoints; H37Rv: p<0.05 at 1 d postinfection, and p<0.01 at other timepoints). To test whether miR-155 affects mycobacterial phagocytosis in macrophages, we transfected RAW264.7 cells with miR-155 mimic or inhibitor, and then challenged with fluorescein Texas red-labeled BCG at an MOI of 10 for 1 h. Flow cytometry was performed to measure mycobacterial phagocytosis in macrophages. Our results showed that neither overexpression nor inhibition of miR-155 had an effect on macrophage-mediated mycobacterial phagocytosis (Fig. 2, G and H).

**Figure 3:**
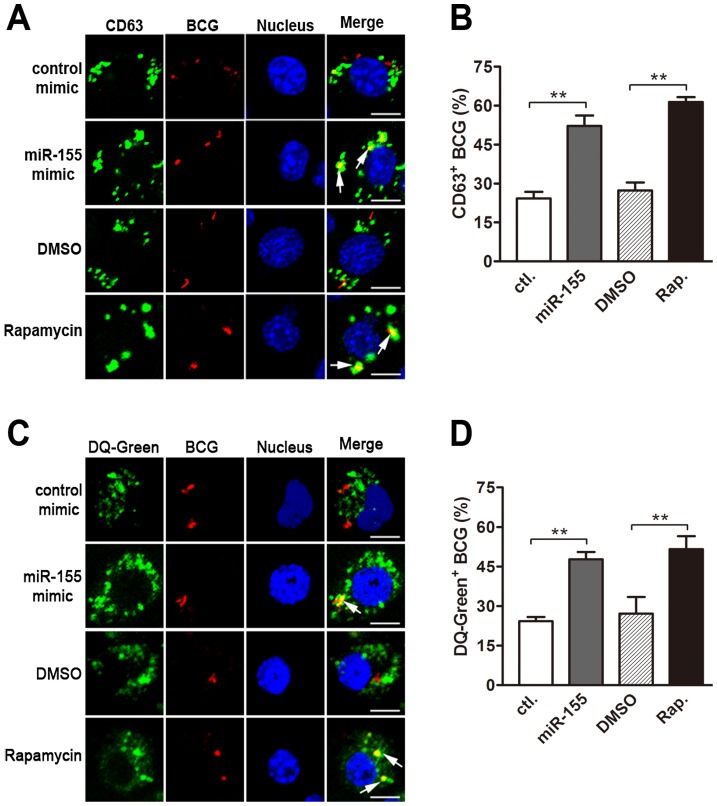
miR-155 promotes mycobacterial phagosome maturation. RAW 264.7 cells were transiently transfected with control or miR-155 mimic for 24 h and then infected with Texas Red-labeled BCG for 1 h. Lysosomes were immunolabeled with CD63 antibody followed by Alexa Fluor 488-conjugated goat anti-rabbit IgG antibody (A), or labeled with a fluorogenic substrate for proteases, DQ-Green (C). The colocalization of BCG with lysosome was detected by confocal microscopy. The percentage of co-localization of BCG with CD63-positive (B) or DQ-Green labeled (D) lysosomes was quantified, respectively. Cells treated with rapamycin were used as a positive control. Arrows indicate the co-localization of BCG with lysosomes; scale bar = 5 µm. Quantification of data are shown as the mean ± SEM of three independent experiments (n = 100 phagosomes). **, p<0.01.

We next explored the mechanisms by which miR-155 promotes the intracellular mycobacterial elimination in macrophages. To this end, we examined the role of miR-155 on the maturation of mycobacterial phagosomes, which is often blocked by mycobacteria to allow their escape from lysosomal bactericidal mechanisms [25]. RAW264.7 cells were challenged with fluorescein Texas red-labeled BCG, and lysosomes were monitored by using immunostaining for lysosomal marker CD63 or staining with DQ Green BSA (DQ-Green, a fluorogenic substrate for proteases). Our results showed that miR-155 enhanced the fusion of BCG phagosomes and lysosomes by nearly two fold, as calculated by the percentage of co-localization of BCG with CD63- or DQ-Green-positive lysosomes (Fig. 3, A–D, both p<0.01). Rapamycin, an inducer of autophagy, was used as a positive control, and treatment increased the percentage of co-localization of BCG with lysosomes by approximately two fold (Fig. 3, A–D, both p<0.01). Taken together, these findings suggest that miR-155 inhibits the survival of intracellular mycobacteria by promoting the maturation of mycobacterial phagosomes.

### miR-155 induces autophagy in macrophages

**Figure 4:**
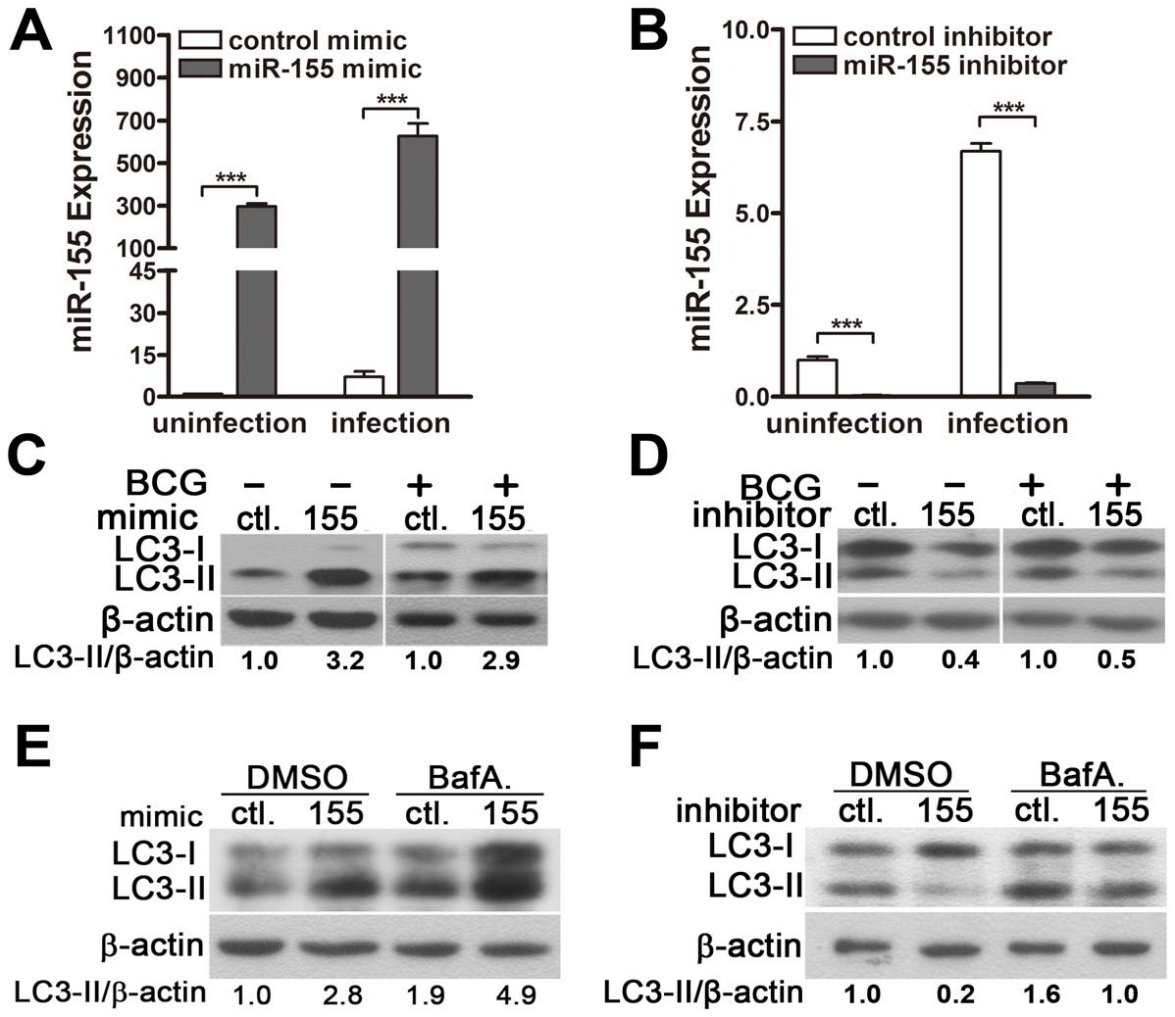
miR-155 induces autophagy in macrophages. (A–D) RAW264.7 cells were transfected with miR-155 mimic (A and C) or inhibitor (B and D) for 24 h and then either left uninfected or infected with BCG. Expression levels of miR-155 were detected by real-time PCR (A and B). The LC3 levels were detected by Western-blot, and the ratios of LC3-II/β-actin were calculated as shown below the representative blot (C and D). (E and F) RAW264.7 cells transfected with miR-155 mimic (E) or inhibitor (F) was incubated with DMSO or bafilomycin A1 (BafA.) at a concentration of 100 nM for 2 h, and then LC3 levels were detected by Western-blot. The ratios of LC3-II/β-actin were calculated as shown below the representative blot. ***, p<0.001.

It has been reported that the induction of autophagy promotes the maturation of mycobacterial phagosomes and the elimination of mycobacteria within infected macrophages [7]. To test the hypothesis that miR-155 induces autophagy in macrophages to enhance the maturation of mycobacterial phagosomes, we evaluated the autophagic activity in RAW264.7 cells using Western-blot and fluorescence microscopy to test the processing of LC3 (conversion from LC3-I to LC3-II) and the number of LC3 puncta, respectively. Real-time PCR data showed that transfection with miR-155 mimic significantly increased the expression level of miR-155 (Fig. 4A), whereas transfection with miR-155 inhibitor markedly decreased miR-155 expression in RAW264.7 cells (Fig. 4B), and with or without BCG challenge. Western-blot results showed that transient transfection with miR-155 mimic activated autophagy in normal uninfected and BCG-infected RAW264.7 cells, as suggested by the increased the amount of LC3-II (Fig. 4C), while transfection with miR-155 inhibitor reduced the amount of LC3-II in RAW264.7 cells before and after BCG challenge (Fig. 4D).

**Figure 5:**
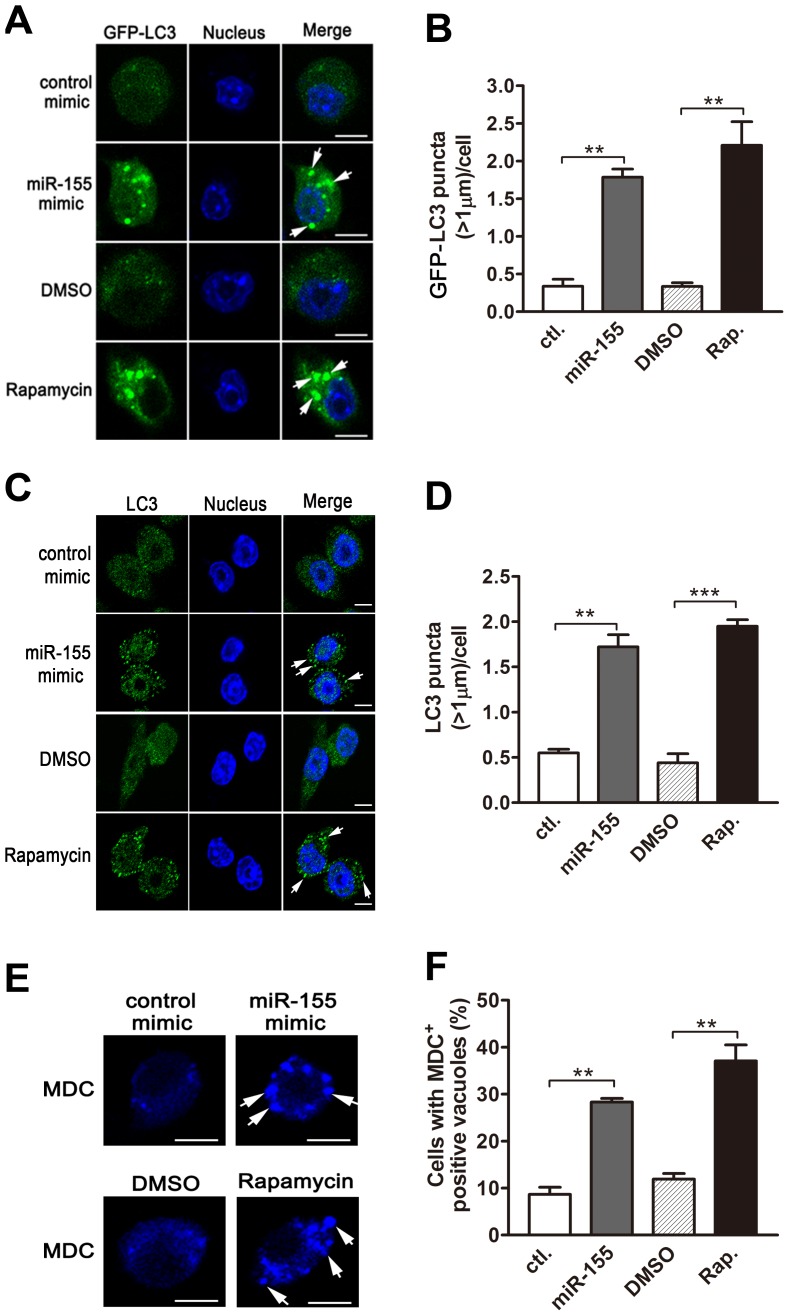
miR-155 promotes autophagosome formation. (A and B) RAW264.7 cells stably expressing GFP-LC3 were transiently transfected with control or miR-155 mimic for 24 h, and GFP-LC3 puncta (>1 µm) were detected by confocal microscopy (A) and quantified (B). RAW264.7 cells were transiently transfected with control or miR-155 mimic for 24 h. Endogenous LC3 was stained with LC3 antibody followed by Alexa Fluor 488-conjugated goat anti-rabbit IgG (Green), and LC3 puncta (>1 µm) were detected by confocal microscopy (C) and quantified (D). (E and F) RAW264.7 cells were transiently transfected with control or miR-155 mimic for 24 h, and then incubated with a specific fluorescent dye MDC (50 µM). The MDC-labeled autophagic vacuoles were detected by confocal microscopy (E) and cells with MDC-positive autophagic vacuoles were quantified (F). Cells treated with rapamycin were used as a positive control. Arrows indicate the GFP-LC3 puncta or autophagic vacuoles labeled with MDC; scale bar = 5 µm. Data are shown as the mean ± SEM of three independent experiments (n = 300 cells). **, p<0.01; ***, p<0.001.

The dynamics of LC3-II accumulation during the process of autophagy depends on both the conversion rate of from LC3-I to LC3-II, and the degradation rate of LC3-II by autolysosomes [12]. To further confirm the role of miR-155 on macrophage autophagy activity, we employed bafilomycin A1, an antagonist of vacuolar H^+^ ATPase, to prevent luminal acidification and autophagosomal cargo degradation. Our results showed that in the presence of bafilomycin A1, transfection with miR-155 mimic in RAW264.7 cells increased the amount of LC3-II (Fig. 4E), while inhibition of miR-155 decreased the amount of LC3-II (Fig. 4F). Furthermore, autophagic activity was examined in RAW264.7 cells stably expressing GFP-LC3, in which the punctate form (type II) of the autophagy marker LC3 can be directly viewed by confocal microscopy. Overexpression of miR-155 led to the redistribution of GFP-LC3 from diffuse to punctate pattern (Fig. 5, A and B, p<0.01). Additionally, the effect of miR-155 on the distribution of endogeneous LC3 was detected by immunofluorescent staining. Results showed that miR-155 promoted the formation of LC3 puncta (Fig. 5, C and D, p<0.01), which supports our Western-blot data. In addition, we confirmed the modulation of autophagy by miR-155 in normal uninfected macrophages by using an autophagic organelle-specific fluorescent dye monodansylcadaverine (MDC). The results of confocal microscopy indicated that the number of MDC-positive autophagic vacuoles was significantly increased in RAW264.7 cells after transient transfection with miR-155 mimic (Fig. 5, E and F, p<0.01). As a potent autophagy inducer, rapamycin markedly augmented the number of LC3 puncta and MDC-positive autophagic vacuoles (Fig. 5, A–F). These data together demonstrate that miR-155 elevates the autophagic response in macrophages.

### miR-155 induces autophagy to eliminate intracellular mycobacteria

**Figure 6:**
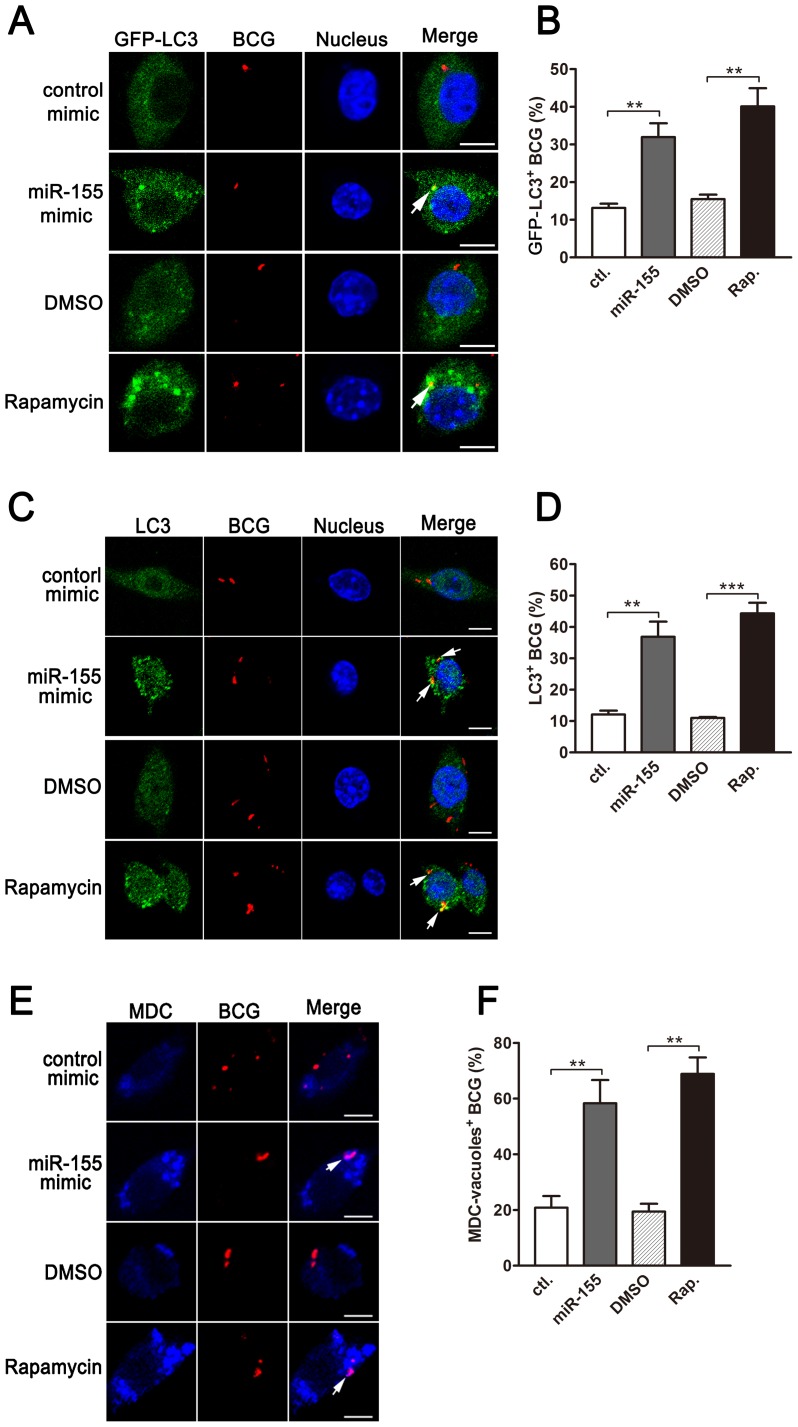
miR-155-induced autophagy promotes the formation of mycobacterial autophagosomes. (A) RAW264.7 cells stably expressing GFP-LC3 were transiently transfected with control or miR-155 mimic and then infected with Texas Red-labeled BCG for 1 h. The co-localization of BCG with LC3 was detected by confocal microscopy. (B) Quantification of the co-localization of BCG with LC3-positive autophagosomes is shown. (C) RAW264.7 cells were transiently transfected with control or miR-155 mimic, and then infected with Texas Red-labeled BCG for 1 h. Endogenous LC3 was stained with LC3 antibody followed by Alexa Fluor 488-conjugated goat anti-rabbit IgG (Green). The co-localization of BCG with endogenous LC3 was detected by confocal microscopy. (D) Quantification of the co-localization of BCG with LC3-positive autophagosomes is shown. (E) After transient transfection with control or miR-155 mimic, RAW264.7 cells were infected with Texas Red-labeled BCG for 1 h, and then were labeled with a specific fluorescent dye MDC (50 µM) for autophagic vacuoles. The co-localization of BCG with MDC-positive autophagic vacuoles was detected by confocal microscopy. (F) Quantification of the co-localization of BCG with MDC-positive autophagosomes is shown. Cells treated with rapamycin were used as a positive control. Arrows indicate the co-localization of BCG with autophagosomes; scale bar = 5 µm. Data are shown as the mean ± SEM of three independent experiments (n = 100 phagosomes). **, p<0.01; ***, p<0.001.

Next, we analyzed the formation of autophagosomes containing BCG in macrophages using confocal microscopy. Our results showed that overexpression of miR-155 enhanced the co-localization of BCG with GFP-LC3-positive autophagosomes in a stable GFP-LC3-expressed RAW264.7 cell line (Fig. 6, A and B, p<0.01). miR-155 overexpression also elevated the co-localization of BCG with endogeneous LC3 autophagosomes (Fig. 6, C and D, p<0.01). Similarly, transfection with miR-155 elevated the co-localization of BCG with MDC-positive autophagic vacuoles in RAW264.7 cells (Fig. 6, E and F, p<0.01). Induction of autophagy with rapamycin markedly increased the co-localization of BCG with LC3 puncta (Fig. 6, A–D) or MDC-positive autophagic vacuoles (Fig. 6, E and F, p<0.01).

**Figure 7:**
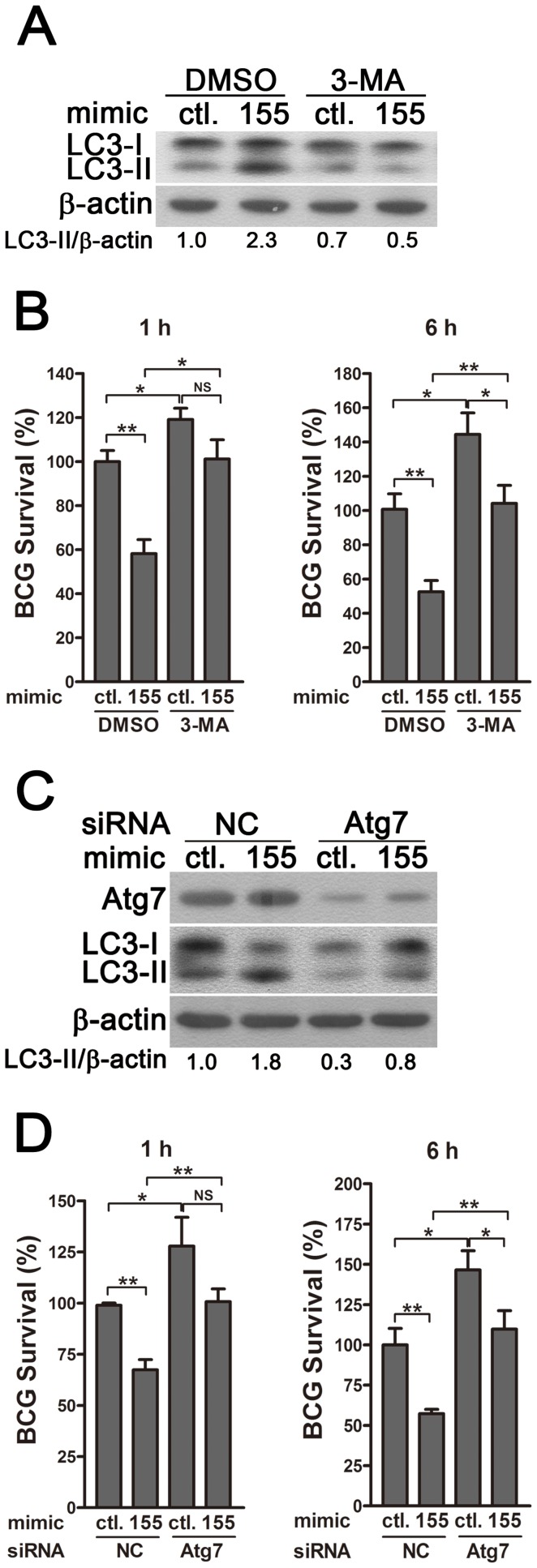
miR-155-induced autophagy promotes the elimination of intracellular mycobacteria. (A and B) RAW 264.7 cells were transiently transfected with control or miR-155 mimic for 24 h, and then incubated with DMSO or 3-MA for 2 h. Protein levels of LC3 were detected by Western-blot in uninfected cells (A). Intracellular mycobacterial viability was determined at the indicated timepoints by CFU assay after BCG challenge for 1 h (B). (C and D) RAW 264.7 cells were transiently co-transfected with control or miR-155 mimic together with a control siRNA or Atg7 siRNA. The expression levels of Atg7 and LC3 were detected by Western-blot (C). Intracellular mycobacterial viability was determined by CFU assay at the indicated time after challenging with BCG for 1 h (D). Values of LC3-II/β-actin ratios are indicated below the representative blot. Data are shown as the mean ± SEM of three independent experiments. *, p<0.05; **, p<0.01; NS, not significant.

To determine whether miR-155 enhances the elimination of intracellular mycobacteria via autophagy, we blocked autophagy by using 3-methyladenine (3-MA). Transfection with miR-155 mimic significantly enhanced autophagy and reduced the viability of BCG in RAW264.7 cells, and these effects were partly reversed by treatment with the autophagy inhibitor 3-MA, as indicated by Western-blot and CFU data (Fig. 7, A and B). RAW264.7 cells also were transfected with specific siRNAs against Atg7, to block the autophagic response. Transfection with Atg7 siRNA dramatically decreased protein expression levels of Atg7 and the amount of LC3-II, indicating the efficacy of Atg7 knockdown and autophagy inhibition (Fig. 7C). More importantly, the miR-155-induced autophagy was markedly inhibited in the RAW264.7 cells co-transfected with Atg7- vs control-siRNA (Fig. 7C). And silencing of Atg7 attenuated the miR-155-mediated mycobactericidal activity, when compared to the control treatment (Fig. 7D). Collectively, these results demonstrate that miR-155 promotes the elimination of intracellular mycobacteria by activating autophagy in macrophages.

### miR-155 post-transcriptionally suppresses Rheb by interacting with its 3′ UTR

**Figure 8:**
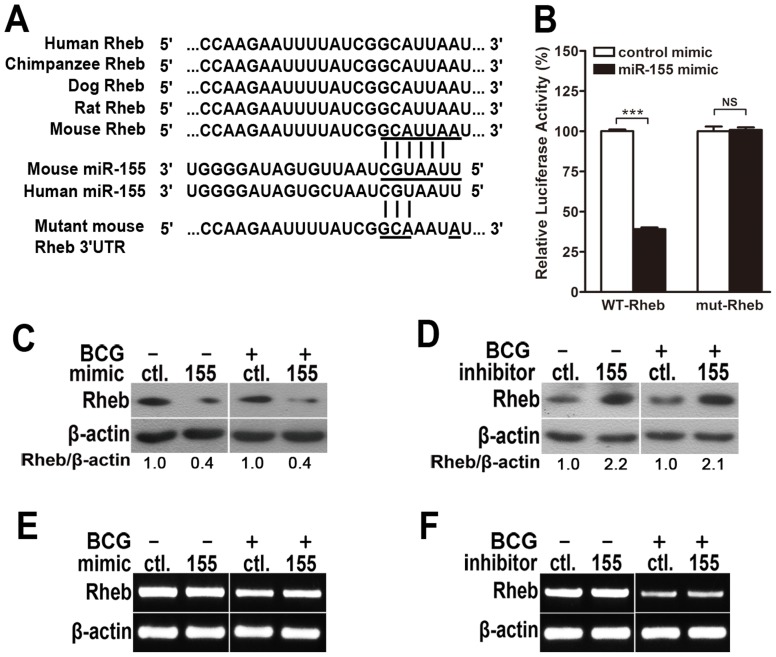
miR-155 post-transcriptionally represses Rheb expression by targeting its 3′UTR. (A) Sequences of mouse and human miR-155 and their predicted interactions with conserved 7-mer 1A miR-155 seeds found within the Rheb 3′UTRs of different species are shown. The sequence of the Rheb 3′UTR seed mutant used for the reporter assays and the predicted disruption of the miR-155 interaction are also shown. (B) RAW264.7 cells were co-transfected with control or miR-155 mimic and a wild-type (WT-Rheb) or mutated Rheb 3′UTR (mut-Rheb) luciferase reporter plasmid and assessed for luciferase activity at 24 h after transfection. Data are shown as the mean ± SEM of three independent experiments. ***, p<0.001; NS, not significant. (C–F) RAW264.7 cells were transfected with miR-155 mimic (C and E) or inhibitor (D and F) for 24 h and either left uninfected or infected with BCG. Protein expression levels of Rheb were detected by Western-blot. Values of Rheb/β-actin ratios are indicated below the representative blot (C and D), and expression levels of Rheb mRNA were detected by RT-PCR (E and F).

To identify the specific target of miR-155 that modulates autophagy, bioinformatics analysis was performed with TargetScan (http://www.targetscan.org/). We found that Rheb, which inhibits autophagy via mTOR, displayed a potential seed match for miR-155 in its 3′-untranslated region (3′UTR) (Fig. 8A). To elucidate whether miR-155 represses Rheb by directly interacting with its 3′UTR, we generated psiCHECK-2-REPORT luciferase constructs containing the 3′UTR of Rheb with the putative miR-155 binding site (WT-Rheb), and used psiCHECK-2-REPORT luciferase constructs containing the 3′UTR of Rheb with a mutation at the putative miR-155 binding site (mut-Rheb, UUA to AAU) as controls (Fig. 8A). RAW264.7 cells were co-transfected with control or miR-155 mimic together with these reporter constructs, followed by assessment of luciferase activity at 24 h after transfection. Overexpression of miR-155 repressed the expression of luciferase fused to the WT Rheb 3′UTR (p<0.001), but failed to repress the expression of luciferase fused to the Rheb 3′UTR containing a mutated miR-155 seed sequence (Fig. 8B).

To explore whether miR-155 represses endogenous Rheb, RAW264.7 cells were transfected with control or miR-155 mimic, and protein levels of Rheb were measured by Western-blot. Rheb protein levels were dramatically decreased in RAW264.7 cells transfected with miR-155- vs control-mimic (Fig. 8C), and increased in RAW264.7 cells after transfection with miR-155- vs control-inhibitor (Fig. 8D). However, in both gain- and loss-of-function studies, miR-155 had no effect on the levels of Rheb mRNA expression (Fig. 8, E and F). Altogether, these results indicate that miR-155 post-transcriptionally represses the expression of Rheb by directly interacting with its 3′UTR seed region.

### miR-155 induces autophagy and promotes the elimination of intracellular mycobacteria by targeting Rheb

**Figure 9:**
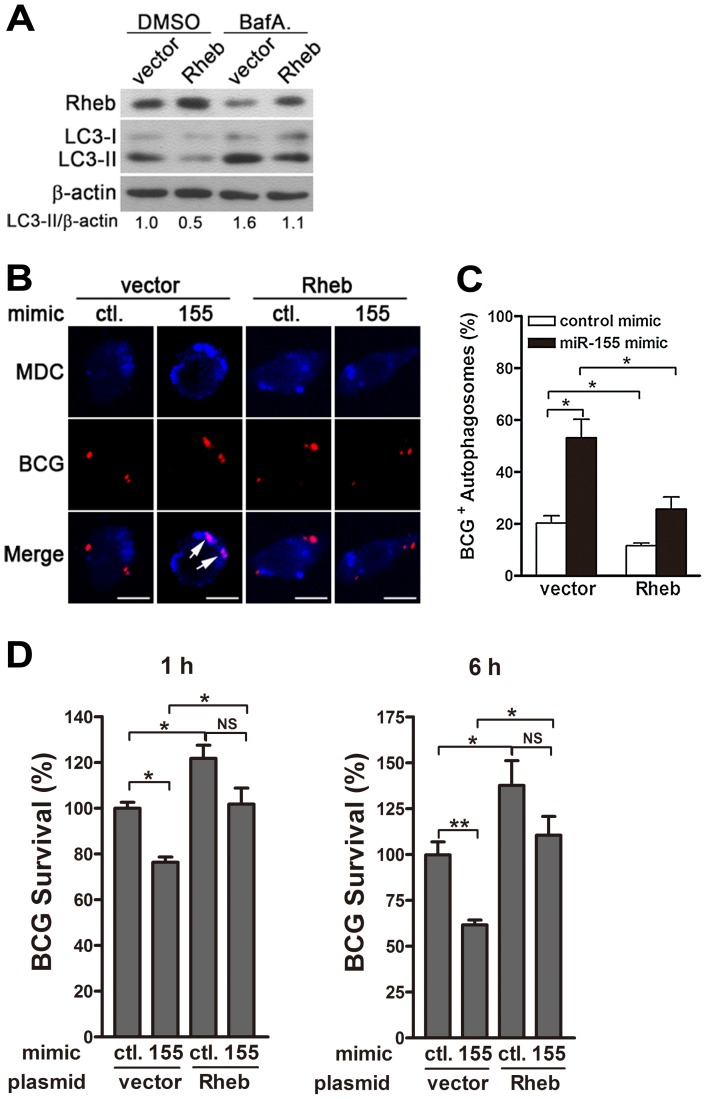
miR-155-induced autophagy promotes the co-localization of BCG with autophagosomes by suppressing Rheb expression. (A) RAW264.7 cells were transfected with a plasmid expressing Rheb for 24 h, and then incubated with DMSO or BafA. for 2 h. The expression levels of Rheb and LC3 were detected by Western-blot. Values of LC3-II/β-actin ratios are indicated below the representative blot. (B and C) RAW264.7 cells were co-transfected with control or miR-155 mimic together with a control plasmid or a plasmid expressing Rheb for 24 h, and then infected with Texas Red-labeled BCG for 1 h. The co-localization of BCG and MDC-labeled autophagosome was detected by confocal microscopy. Arrows indicate Quantification of BCG co-localization with autophagosomes described in B is shown (C). (D) RAW264.7 cells were co-transfected with control or miR-155 mimic together with a control plasmid or a plasmid expressing Rheb for 24 h. Cells were infected with BCG for 1 h, and washed for three times to remove extracellular mycobacteria. Intracellular mycobacterial viability was determined by CFU assay at the indicated timepoints. Data are shown as the mean ± SEM of three independent experiments. *, p<0.05; **, p<0.01; NS, not significant.

The next series of experiments were designed to explore whether miR-155 promotes autophagy-induced bacterial elimination by targeting Rheb. We first tested the effect of Rheb on autophagy in RAW264.7 cells. Transient transfection with a plasmid expressing Rheb effectively increased protein levels of Rheb and downregulated the amount of LC3-II (Fig. 9A). Furthermore, blocking autophagy flux with bafilomycin A1 effectively inhibited the degradation of LC3-II, and in the presence of bafilomycin A1, overexpression of Rheb also decreased the amount of LC3-II, when compared with treatment with control plasmid (Fig. 9A), indicating that Rheb overexpression dramatically inhibits autophagy in macrophages. To further determine whether miR-155 enhances the elimination of intracellular mycobacteria by targeting Rheb, RAW264.7 cells were co-transfected with miR-155 mimic and Rheb-expressing plasmid, and then confocal microscopy and CFU assay were performed to detect the maturation of mycobacterial autophagosomes and intracellular bacterial load, respectively. Confocal microscopy results showed that overexpression of miR-155 enhanced the co-localization of mycobacterial phagosomes and MDC-positive autophagic vacuoles in RAW264.7 cells after co-transfection with a control vector (Fig. 9, B and C, p<0.05), suggesting that miR-155 promotes the formation of mycobacterial autophagosomes. However, miR-155-mediated formation of mycobacterial autophagosome in RAW264.7 cells was abrogated by co-transfection with Rheb-expressing plasmid (Fig. 9, B and C, p<0.05). Furthermore, CFU assay results indicated that overexpression of miR-155 significantly decreased the bacterial load of intracellular BCG by 25% and 40% at 1 h and 6 h postinfection, respectively (Fig. 9D, both p<0.01), whereas Rheb overexpression significantly inhibited miR-155-mediated mycobactericidal activity in RAW264.7 cells (Fig. 9D, p<0.05). Together, these results suggest that miR-155 promotes autophagy-mediated elimination of intracellular mycobacteria by targeting Rheb.

## Discussion

Autophagy has been demonstrated to play an essential role in the host immune response against mycobacterial infection. Nonetheless, the molecular basis involved in autophagy-mediated mycobacterial clearance remains largely unclear. Here we report a novel role of miR-155 in regulating autophagy and mycobacterial elimination in macrophages by targeting Rheb, which may provide a better understanding of the host anti-mycobacterial response.

Emerging evidence has shown that miR-155 is mainly expressed in activated macrophages [26], dendritic cells [27], B [28] and T lymphocytes [29]. miR-155 expression is up-regulated by a variety of inflammatory mediators and pathogens [26], [30]. Our study shows that miR-155 expression is enhanced both *in vivo* and *in vitro* after mycobacterial infection, which is consistent with previous study showing the miR-155 induction in response to avirulent *M. smegmatis* [22] and *M. bovis* BCG challenge [31]. Recent studies have revealed that *M. tuberculosis* purified protein derivative (PPD) induces a high expression of miR-155 in peripheral blood mononuclear cells (PBMCs) from active TB patients vs healthy donors [32]. These data implicate a potential correlation of miR-155 with mycobacterial infection.

Studies have revealed that miR-155 participates in various biological processes including oncogenicity, haemotopoiesis and inflammatory responses [33]. *In vivo* studies using miR-155 deficient mice have demonstrated that miR-155 is required for normal immune function of T and B lymphocytes as well as dendritic cells [34]. It is also reported that miR-155 promotes the development of T helper 1 (Th1) and Th17 cell subsets [35], and attenuates the Th2 cell response by targeting c-Maf, a potent transactivator of the IL-4 promoter [36]. Moreover, miR-155 contributes to the classical activation (M1 polarization) of macrophages and iNOS expression by targeting CCAAT/enhancer binding protein β (CEBPβ), a hallmark of alternatively activated (M2) macrophage [37]. These studies together implicate a potent role of miR-155 in the cellular immune response, which has been demonstrated as the major arm of host anti-mycobacterial defense.

As one of the most important cell types in the anti-mycobacterial immunity, macrophages function as the predominant responder cell against *M. tuberculosis* infection. The cells uptake and kill mycobacteria and by initiating an inflammatory response, but also provide a preferred hiding and replication site for mycobacteria [38]. Therefore, precisely modulating of macrophage activity is crucial in the protective immunity against mycobacterial infection.

The exact role of miR-155 in macrophages during mycobacterial infection has yet to be elucidated. Studies have demonstrated that miR-155 enhances TNF production in human macrophages in response to mycobacteria [22], and TNF in turn elevates macrophage activity to eliminate intracellular mycobacteria [39], [40], indicating that miR-155 might be required in the macrophage-mediated immune defense against infection. In the present study, RAW264.7 cells were infected with mycobacteria at an MOI of 10 for 1 h, and then incubated for another 1 h, 6 h, 1 d, 2 d, and 3 d, after removing the extracellular bacteria. Our results demonstrate that miR-155 promoted the maturation of mycobacterial phagosome and decreased the survival of intracellular BCG at all tested timepoints, indicating that miR-155 promoted macrophage-mediated bacterial elimination. However, it is reported that in some cases, miR-155 may display a different role in modulating mycobacterial survival. For example, Ghorpade et al. reported that overexpression of miR-155 in RAW264.7 cells promoted apoptosis and increased intracellular bacterial load at 96 h postinfection (including a 24 h infection at an MOI of 10 plus a 72 h postinfection incubation) [31]. Ghorpade's observation is inconsistent with others showing that induction of apoptosis in infected host cells reduces the viability of intracellular mycobacteria [41], but could be explained by Lee's findings showing that high numbers of intracellular mycobacteria trigger macrophage necrosis that could promote mycobacterial survival [42]. In Ghorpade's study, the percentage of viable cells was markedly decreased to 10% after transfection with miR-155 overexpressing plasmid plus BCG infection, thus greatly increasing the actual MOI during infection.

Autophagy has been proved to be a crucial element of the innate immune response against intracellular pathogens, including mycobacteria [43]. In the present study, we demonstrate that miR-155 induces the processing of LC3 and the accumulation of LC3 puncta in both BCG-challenged and unchallenged RAW264.7 cells, which indicates that miR-155 accelerates the autophagic response in macrophages and reduces intracellular bacterial load. Moreover, we observed that inhibition of autophagy with 3-MA or silencing Atg7 reduced miR-155-mediated autophagy and bacterial killing. In addition, we found that in control mimic treated RAW264.7 cells, treatment with 3-MA dramatically increased the survival of BCG at 6 h, though the reduction of the level of LC3-II is modest. This may be explained by other reports showing that 3-MA promotes the survival of mycobacteria by suppressing nitric oxide production [44].

Autophagy not only elevates the delivery of mycobacterial phagosomes for mycobacterial killing [7], but also enhances the presentation of mycobacterial antigens (e.g., Ag85B) to induce a protective CD4^+^ T lymphocyte response [45]. Both *in vivo* and *in vitro* studies have shown that induction of autophagy effectively increases the intracellular killing of *M. tuberculosis* [46], suggesting that targeting autophagy may be a potential therapeutic strategy for TB treatment. Interestingly, our results showed that inhibition of autophagy by 3-MA or Atg7 siRNA attenuated, but not fully blocked the miR-155-mediated mycobactericidal activity at 6 h postinfection (Fig. 7, B and D), indicating that in addition to autophagy, other alternative mechanisms may be involved in the miR-155-mediated bacterial elimination. Studies have demonstrated that macrophages also employ an oxygen-dependent system to control intracellular pathogens [47], [48], [49]. Our unpublished data showed that overexpression of miR-155 did not influence iNOS expression or nitric oxide production, but slightly enhanced the production of reactive oxygen species in BCG-infected RAW264.7 cells (data not shown), suggesting that miR-155 may exert antimycobacterial function partially through enhancing ROS production.

Studies have demonstrated that miR-155 implements different functions in various physical, pathological and experimental conditions by repressing distinct targets, including, but not limited to, TGF-β activated kinase 1/MAP3K7 binding protein 2 (TAB2) [27], suppressor of cytokine signaling 1 (SOCS1) [50], PU.1 [51], CEBPβ [52], Src homology-2 domain-containing inositol 5-phosphatase 1 (SHIP-1) [53], myeloid differentiation primary response gene 88 (MyD88) [54], phosphoinositide 3-kinase (PI3K) p85 [55]. Most of these miR-155 targets are involved in modulating inflammatory response. Among these targets of miR-155, PI3K p85 plays an important role in regulating autophagy. However, our unpublished data from luciferase assay and Western blot demonstrated that miR-155 did not bind to the 3′UTR of p85, and had no influence on p85 expression in RAW264.7 cells (data not shown), which differs from other reports showing that miR-155 targets p85 in human B-cell lymphoma cells [55]. Studies have demonstrated that different targets may be involved in different systems [33], [56]. For example, miR-125b is reported to promote hepatocellular carcinoma cell apoptosis by targeting Bcl-2, an anti-apoptotic protein [57], while miR-125b inhibits apoptosis of Hela cells and human immortalized myelogenous leukemia K562 cells by targeting pro-apoptotic proteins, Bak1, Mcl1 and p53 [58].

In the present study, we identified that Rheb is a novel target for miR-155. Rheb functions as a negative regulator of autophagy, by directly interacting with mTOR [17] and increasing the mTOR activity [59]. Rheb activity is regulated by upstream kinases, such as Akt, AMP-activated protein kinase, and glycogen synthase kinase-3β, while little is known regarding the mechanism involved in modulating Rheb expression. It is reported that Rheb expression is increased in hepatocytes after Hepatitis C virus infection [60], but reduced in RAW264.7 cells after treatment with hydrogen peroxide [61]. Our study indicates that miR-155 post-transcriptionally down-regulates the expression level of Rheb, thus activating autophagy in macrophages. Moreover, CFU results suggest that overexpression of Rheb reduces the ability of miR-155 to induce intracellular bacterial clearance, indicating that Rheb is the major target of miR-155 in modulating the autophagic response and mycobacterial elimination in macrophages.

A recent study showed that *Shigella* infection results in sustained amino acid starvation and mTOR inhibition, thereby triggering the autophagy response in host cells for innate host defense, whereas *Salmonella* only induces a transcient amino acid starvation, favoring mTOR reactivation and leading to autophagy escape [62]. These findings reflect the intimate link between host metabolism and autophagic response against invading pathogens. Our study demonstrates that mycobacteria-induced miR-155 targets Rheb, a positive regulator of mTOR signaling, which is crucial in sensing and responding to cellular nutrients and stress [63], suggesting that miR-155 may have a potential role in modulating the metabolic signaling in host cells.

Collectively, the present study demonstrates that the miR-155 is induced by mycobacterial infection, and promotes autophagy in macrophages by targeting Rheb, conferring protection against infection with intracellular mycobacteria. Our study unravels an important role of miR-155 in autophagy regulation and mycobacterial elimination, which may provide useful information for developing potential therapeutic interventions against tuberculosis.

## Materials and Methods

### Ethics statement

All animal experiments were performed in accordance with the National Institutes of Health Guide for the Care and Use of Laboratory Animals, and the experimental procedures were approved by the Medical Ethics Committee of

Sun Yat-sen University Zhongshan School of Medicine and the Biosafety Management Committee of Sun Yat-sen University (No. 2012-33).

### Mice and reagents

Eight-week-old BALB/c and C57BL/6 (B6) mice were purchased from Sun Yet-sen University Animal Supply Center. Middlebrook 7H9 broth medium and Middlebrook 7H10 agar were purchased from BD Difco Laboratories (Sparks, MD). The CD63 antibody was obtained from Santa Cruz Biotechnology (Santa Cruz, CA). The LC3 antibody was purchased from Novus Biologicals (Littleton, CO). Antibodies against Atg7 and Rheb were obtained from Cell Signaling Technology (Beverly, MA). The β-actin antibody, MDC, bafilomycin A1, 3-methyladenine, rapamycin and DMSO vehicle control (0.2%) were obtained from Sigma-Aldrich (St. Louis, MO). The Texas Red and DQ-Green dyes were purchased from Invitrogen (Carlsbad, CA).

### Cells and bacterial culture

Murine macrophage-like RAW264.7 cells (ATCC; TIB-71) were cultured in DMEM supplemented with 10% fetal bovine serum (FBS) and 100 U/ml penicillin, 100 µg/ml streptomycin (GIBCO, Invitrogen). BMDMs were prepared by culturing bone marrow from the femurs and tibiae of 6- to 8-week old B6 mice in DMEM containing 10% FBS, 2 mM L-glutamine, 1 mM sodium pyruvate, 100 U/ml penicillin, 100 µg/ml streptomycin, and 10% L929 conditioned medium. Non-adherent cells were removed after 24 h and cultured for 7 days. *M. bovis* BCG strain 19015, *M. tuberculosis* H37Ra strain 25177 and *M. tuberculosis* H37Rv strain 25618 were purchased from the American Type Culture Collection (ATCC), and mycobacteria were grown in Middlebrook 7H9 broth medium or on 7H10 agar plates supplemented with OADC and cultured in a standard tissue culture incubator at 37°C with an atmosphere of 5% CO_2_ and 95% air.

### Infection of mice

*M. tuberculosis* H37Rv was homogenized to generate a single cell suspension. BALB/c mice were intraperitoneally injected with *M. tuberculosis* H37Rv (5×10^6^ CFU/per mouse). After 6 weeks, lungs were removed from sacrificed mice. Total RNA was isolated using TRIzol reagent (Invitrogen) according to the manufacturer's recommendations. Animal experiments were performed in accordance with the approval of the Scientific Investigation Board of Sun Yat-sen University (Guangdong, China).

### Plasmids and siRNA

pEX-GFP-LC3 was from Addgene (#24987), which was deposited by Isei Tanida [64]. The 3′UTR and cDNA sequence of mouse *Rheb* was amplified by reverse transcription-PCR and cloned into psiCHECK-2 (Promega, Madison, WI) and pMSCV-neo (Clontech, Mountain View, CA), respectively. The mutation in the 3′UTR of mouse *Rheb* was generated with the QuikChange Site-Directed Mutagenesis Kit from Stratagene (La Jolla, CA) following the manufacturer's protocol. Negative control (NC) siRNA was purchased from Invitrogen. Atg7 siRNA was purchased from Dharmacon/Thermo Fisher Scientific (Waltham, MA).

### Transient transfection

RAW264.7 cells (at approximately 50% confluence) were transiently transfected with 30 nM control or miR-155 mimic (Applied Biosystems, Foster City, CA); 50 nM control or miR-155 LNA-inhibitor (EXIQON, Vedbaek Denmark); or 1.6 µg plasmid; or 40 pmol siRNA, using Lipofectamine 2000 (Invitrogen) according to the manufacturer's instructions.

### Colony-Forming Unit (CFU) assay

RAW264.7 cells were infected with *M. bovis* BCG or *M. tuberculosis* H37Ra or H37Rv at an MOI of 10. After 1 h incubation at 37°C, the infected cells were washed extensively with PBS to remove extracellular mycobacteria, and the infected cells were incubated for another 1 h, 6 h, 1 d, 2 d, or 3 d, and then lysed in 1 ml of distilled water. Quantitative culturing was performed using 10-fold serial dilutions. Aliquots of each dilution were inoculated in triplicate on Middlebrook 7H10 agar plates with OADC. Plates were incubated for 3 weeks, and colonies were counted.

### Phagocytosis assays

Phagocytosis was assayed by flow cytometry, as described by others [65]. Briefly, BCG was incubated with Texas Red (Invitrogen) at room temperature for 2 h, protected from light, and then gently rinsed with PBS. Then RAW264.7 cells were challenged with Texas Red -labeled BCG at an MOI of 10. After 1 h incubation, cells were washed three times with cold PBS and centrifuged to remove extracellular bacteria, and then analyzed by flow cytometry using a Beckman Coulter EPICS XL/MCL (Beckman Coulter Inc., Fullerton, CA) instrument.

### Luciferase reporter assays

RAW264.7 cells (at approximately 50% confluence) were cultured in 24-well plates one day prior to transfection. psiCHECK-2 luciferase reporter plasmids (Promega, Madison, WI) containing either a wild-type or mutated Rheb 3′UTR were co-transfected with control or miR-155 mimic into RAW264.7 cells with Lipofectamine 2000 (Invitrogen). Cells were harvested 24 h later, and luciferase activity was assessed with the Dual-Luciferase Reporter Assay System (Promega) following the manufacturer's protocol.

### RAW264.7 GFP-LC3 stable cell line

RAW264.7 cells were transfected with pEX-GFP-LC3 using Lipofectamine 2000 according to manufacturer's instructions. Stable transfectants were selected for 3 weeks with 1 mg/ml G418 and maintained in 0.2 mg/ml G418. The transfectants were confirmed by Western-blot, and the formation of LC3 puncta in response to rapamycin was assessed by fluorescence microscopy (data not shown).

### RT-PCR and real-time PCR analysis

Total RNA was isolated using TRIzol reagent (Invitrogen) according to the manufacturer's recommendations. For miRNA, the expression levels of miR-155 were detected using a TaqMan microRNA kit (Applied Biosystems) and normalized to small nuclear RNA (Rnu6). For mRNA, first-strand cDNA synthesis was performed using RevertAid First Strand cDNA Synthesis Kit (Thermo Fisher Scientific, Waltham, MA). For RT-PCR, the expression of Rheb was assessed by PCR amplification using a standard protocol. Amplified products were fractionated by 1% agarose gel electrophoresis and visualized by ethidium bromide staining. The primer sequences used for PCR were: Rheb, 5′- ATGCCTCAGTCCAAGTCCCGGA AG-3′ (forward) and 5′- TCACATCACCGAGCACGAAGA -3′ (reverse); β-actin, 5′-GATTACTGCT CTGGCTCCTAGC -3′ (forward) and 5′- GACTCATCGTAC TCCTGCTTGC -3′ (reverse).

### Western-blot analysis

Cells were washed three times with ice-cold PBS and then lysed in lysis buffer containing 1 mM phenylmethylsulfonyl fluoride, 1% (vol/vol) protease inhibitor cocktail (Sigma), and 1 mM DTT. Equal amounts (20 µg) of cell lysates were resolved by SDS-PAGE and then transferred to PVDF membranes. Membranes were blocked in 5% non-fat dry milk in PBST and incubated overnight with the respective primary antibodies at 4°C. The membranes were incubated at room temperature for 1 h with appropriate HRP-conjugated secondary antibodies and visualized with Plus-ECL (PerkinElmer, Shelton, CA) according to the manufacturer's protocol.

### Confocal microscopy

For immunofluorescence experiments, cells were grown on collagen-precoated glass coverslips in 24-well plates. Cells were transiently transfected with control or miR-155 mimic (30 nM) for 24 h and then infected with Texas Red-labeled BCG (MOI 10) for 1 h. Cells were fixed with 4% paraformaldehyde followed by membrane permeabilization using 0.2% Triton X-100. Cells were blocked with 5% BSA and incubated with primary and then secondary antibodies before mounting. In fluorescence experiments, RAW264.7 GFP-LC3 cells were transfected with control or miR-155 mimic using Lipofectamine 2000 and either left uninfected or infected with Texas Red-labeled BCG (MOI 10) for 1 h. MDC staining was performed by adding MDC (50 µM) to cells and incubating at 37°C for 30 min. The cells were fixed in 4% paraformaldehyde for 10 min and viewed by confocal microscopy (Zeiss Axiovert, LSM710).

### Statistical analysis

Unpaired Student's t test or one-way analysis of variation was used to determine the significance of the results from real-time RT-PCR experiments and CFU assays, the quantification of GFP-LC3 puncta, MDC-positive autophagic vacuoles and the co-localization of BCG with lysosomes or autophagosomes. Data were considered statistically significant at p<0.05.

### Accession numbers of genes mentioned in the manuscript

microRNA-155: [ *Mus musculus*]. NCBI Gene ID: 387173.

*Rheb*: [*Mus musculus*]. NCBI Gene ID: 197744.

*Atg7*: [*Mus musculus*]. NCBI Gene ID: 74244.
